# RACIAL-ETHNIC VARIATION IN MULTIMORBIDITY PATTERNS AND HEALTHCARE SERVICES AMONG OLDEST-OLD PATIENTS

**DOI:** 10.1093/geroni/igac059.2870

**Published:** 2022-12-20

**Authors:** Jinmyoung Cho, Gelareh Rahimighazikalayeh, Heather Allore

**Affiliations:** Baylor Scott &White Research Institute, Temple, Texas, United States; Baylor Scott & White Research Institute, Dallas, Texas, United States; Yale University, New Haven, Connecticut, United States

## Abstract

Adults aged 85 years and older (“oldest-old”) are perceived as survivors resilient to age-related risk factors. Although considerable heterogeneity has been often observed in this population, less is known about the unmet needs in health and healthcare service utilization for diverse patients in healthcare systems. We examined racial-ethnic variation in patterns of multimorbidity associated with emergency department (ED) and clinic visits among oldest-old patients with multiple chronic conditions (MCCs). Administrative and clinical data from an integrated healthcare system for five years included 25,801 oldest-old patients with MCCs. Hierarchical cluster analysis identified patterns of MCCs by four racial-ethnic groups (White, Black, Hispanic, & Other). Clusters associated with ED and clinic visits were analyzed using generalized estimation equations. The average of 5.79 (±2.79) MCCs ranging from 5.36 (±2.61) for Other to 5.82 (±2.79) for Hispanic patients at baseline decreased over time. Hypothyroidism, Alzheimer’s Disease and related dementia, bone-and-joint, metabolism syndrome, and pulmonary-vascular clusters were commonly observed across the groups. Unique cluster patterns were identified among Black patients (e.g., renal diseases were grouped with metabolic syndrome cluster). While almost all clusters were significantly associated with ED and clinic visits among White patients, distinctive clusters were significantly related to ED and clinic visits among Hispanic patients (e.g., bone–and-joint cluster grouped with renal diseases was significantly associated with ED [RR=1.36, p < .0001] and clinic [RR=1.39, p < .0001] visits, respectively). Patterns of multimorbidity and its significant association with healthcare service utilization varied by race-ethnicity. Findings suggest a need for culturally tailored care management within integrated healthcare systems.

